# Catalase-Knockout Complements the Radio-Sensitization Effect of Titanium Peroxide Nanoparticles on Pancreatic Cancer Cells

**DOI:** 10.3390/molecules30030629

**Published:** 2025-01-31

**Authors:** Winda Tasia, Amane Washio, Koki Yamate, Kenta Morita, Yutaro Mori, Prihardi Kahar, Ryohei Sasaki, Chiaki Ogino

**Affiliations:** 1 Department of Chemical Science and Engineering, Graduate School of Engineering, Kobe University, Kobe 658-8501, Hyogo, Japan; wind013@brin.go.id (W.T.); kmorita@port.kobe-u.ac.jp (K.M.); yutaro.mori@hawk.kobe-u.ac.jp (Y.M.); pri@port.kobe-u.ac.jp (P.K.); 2 National Research and Innovation Agency (BRIN), Bogor 16911, West Java, Indonesia; 3 Division of Radiation Oncology, Graduate School of Medicine, Kobe University, Kobe 650-0017, Hyogo, Japan; rsasaki@med.kobe-u.ac.jp

**Keywords:** titanium oxide nanoparticles, catalase, radio-sensitizer, ROS, hypoxia

## Abstract

In previous studies, titanium peroxide nanoparticles (PAA-TiOx NPs) with surfaces functionalized using polyacrylic acid (PAA) and hydrogen peroxide (H_2_O_2_) demonstrated a synergistic effect when combined with X-ray irradiation. The combination generated H_2_O_2_ and reactive oxygen species (ROS) that enhanced the irradiation efficacy. In the present study, we examined the relationship between catalase and PAA-TiOx NPs sensitization to X-ray radiation because catalase is the primary antioxidant enzyme that converts H_2_O_2_ to water and oxygen. Catalase-knockout PANC-1 (dCAT) cells were generated using the CRISPR/Cas9 system, which was confirmed by the suppression of catalase expression in mRNA and protein levels that resulted in an 81.7% decrease in catalase activity compared with levels in wild-type cells. Catalase deficiency was found to increase the production of ROS, particularly in hypoxia. Also, the combination of PAA-TiOx NPs and X-ray 5 Gy resulted in a 7-fold decrease in the survival fraction (SF; *p* < 0.01) of dCAT cells compared with rates documented in wild-type cells. Interestingly, the combination treatment with X-ray 3 Gy in dCAT cells resulted in an SF similar to that observed in wild-type cells treated with the same combination but at a higher radiation dose (5 Gy). These results suggest that a strategy of catalase inhibition could be used to establish an advanced combination treatment of PAA-TiOx NPs and X-ray irradiation for pancreatic cancer cells.

## 1. Introduction

Radiation therapy (radiotherapy) is an important modality that is widely used in cancer treatment [1]. Twenty million new cancer diagnoses were estimated in 2022. According to these data, the projection for new cancer diagnoses by the year 2050 could be as high as 33.1 million. The data indicate that from 2022 to 2050, 6.5 million individuals could require radiotherapy at an estimated use rate of 50% [2]. Radiotherapy utilizes ionizing radiation, which at the cellular level results in deoxyribonucleic acid (DNA) damage and impaired cell replication. Free radicals are also involved in this process. They are more sensitive to cancer cells than normal cells because normal cells have DNA-repair mechanisms that are more efficient in counteracting the damage. Despite this, consideration is necessary to achieve the maximum possible dose for the best therapeutic effect while minimizing the side effects on surrounding normal cells [1,3].

The application of high-dose radiation is limited by the side effects on normal cells [4,5]. Thus, fractionated radiotherapy is applied to provide normal cells time to recover between treatments because healthy cells possess better damage-repair capability than tumor cells [6,7]. Amifostine, a cytoprotective agent, has also been studied for radioprotective effects on a broad range of normal tissues [8]. Currently, amifostine is mainly used to reduce side effects, acute, and late toxicity by radiotherapy in the head and neck. It typically reduces mucositis, dry mouth, and taste disorders [9]. Concern is placed on radiation-induced damages, and polycysteine is studied to address the adverse effects of amifostine. It is a new type of radio-protector, and the preliminary results showed similar efficacy to amifostine but with a better safety profile [10].

However, radiotherapy resistance caused by low oxygen conditions is another limitation that has become a concern [11,12,13]. One approach to resolving this problem is employing chemical radio-sensitizers [14], such as tirapazamine [15] and nimorazole [16]. Besides that, nanoparticle-based radio-sensitizers are continuously being studied due to their particular features, which include enhanced permeability and a retention effect, which promotes nanoparticles (NPs) to localize in tumor tissues [17]. High-atomic number metal-based NPs primarily accommodate radiation amplification to generate secondary electrons and reactive oxygen species (ROS), which elevate localized damage [18]. However, the bottleneck of nanoparticle-based radio-sensitizers in the clinical experience remains in the heterogeneity among human tumors that correlates to enhanced permeability and a retention effect. Additionally, while nanoparticle-based radio-sensitizers are administered intratumorally, the intratumoral variability in human tumors cannot be detected as rapidly as in animal models [17], which may complicate the clinical applications. It is encouraging that hafnium oxide NPs (NBTXR3) are currently undergoing clinical trials as a radio-enhancer for pancreatic ductal adenocarcinoma. The report of the first patient experience demonstrated the initial feasibility of endoscopic ultrasound-guided intratumoral injection of NBTXR3 activated by radiotherapy for patients with unresectable pancreatic cancer. The patient received NBTXR3 without any acute adverse events [19].

Investigating radio-sensitizers is particularly important for pancreatic cancer treatment because pancreatic cancer desmoplasia causes tumor hypoxia, which negatively affects the susceptibility of the cancer cells to radiation [20,21]. In addition, radiation resistance is one of the factors contributing to the poor prognosis of pancreatic cancer cases [20,22], which leads to the meager 5-year survival rates [23,24]. In order to address this challenge, we have been developing titanium-based NPs as a potential radio-sensitizer that continuously releases hydrogen peroxide (H_2_O_2_) into the liquid phase of a dispersion. Modification of titanium dioxide (TiO_2_) NPs with polyacrylic acid (PAA) and H_2_O_2_ were established as titanium peroxide NPs (PAA-TiOx NPs) [25]. The combination treatment of PAA-TiOx NPs and X-ray irradiation has shown remarkable results in inhibiting tumor growth compared with a regimen of irradiation alone in mice xenograft pancreatic tumor models. Neither apparent cytotoxicity nor weight loss has been observed in mice until 43 days post-irradiation [26]. In another study, the tissue distribution of PAA-TiOx NPs was monitored using pancreatic cancer-xenografted mice. After the intravenous injection, PAA-TiOx NPs had remarkably pooled in tumors via the enhanced permeability and retention effect, although these had accumulated in the livers [27]. It was revealed that PAA-TiOx NPs followed by X-ray irradiation generate intracellular ROS, predominantly H_2_O_2_ and hydroxyl radicals (OH·) [26], but, notably, the generation of H_2_O_2_ by PAA-TiOx NPs was defined as the major factor in the radio-sensitization effect of the NPs, compared with gold NPs [28].

Since catalase is a key antioxidant enzyme in converting H_2_O_2_ to oxygen and water [29], in this study, we specifically focused on exploring the relationship between catalase and the combination treatment of PAA-TiOx NPs and X-ray radiation. We hypothesized that modulating catalase expression and activity could increase the therapeutic effects of the combination treatment. This approach extends our previous works on improving X-ray irradiation efficacy using PAA-TiOx NPs as a radio-sensitizer. Furthermore, we provide new insights into the in vitro study of the combination treatment in pancreatic cancer cells in hypoxic conditions, which were not addressed in prior research.

## 2. Results and Discussions

### 2.1. Characterization of PAA-TiOx NPs

The particle size distribution of synthesized PAA-TiOx NPs was determined using dynamic light scattering. The average hydrodynamic diameter was found to be 94.3 ± 1.2 nm with a polydispersity index (PdI) of 0.15, indicating a uniform population (Figure S1A and Table S1). The size was consistent with previously reported studies that exploited the enhanced permeability and retention effect [26,27]. PAA-TiOx NPs were established by modifying TiO_2_ NPs with PAA and H_2_O_2_. Modification with PAA aimed for high dispersibility in a saline solution by shifting the isoelectric point and surface charge of TiO_2_ NPs. Before the modification, TiO_2_ NPs would not disperse in purified water and ionic solution because the isoelectric point is approximately at a neutral state. After the modification, PAA-TiO_2_ NPs obtained dispersibility in neutral and ionic solutions, even after the absorption of H_2_O_2_. Thus, PAA-TiOx NPs could achieve stability in the blood [25]. The surface of PAA-TiOx NPs was negatively charged at −31.3 ± 0.4 mV (Figure S1B and Table S1), which agrees with the results of a study by Morita et al. [30]. This anionic charge on the surface may lead to the dispersion of PAA-TiOx NPs. In addition, the merits of adsorbed H_2_O_2_ in PAA-TiOx NPs included a longer retention time and continuous availability compared with the use of H_2_O_2_ alone. Further characterization details are documented in prior research [25].

### 2.2. Generation of Catalase-Knockout PANC-1 (dCAT) Cells

**Figure 1:**
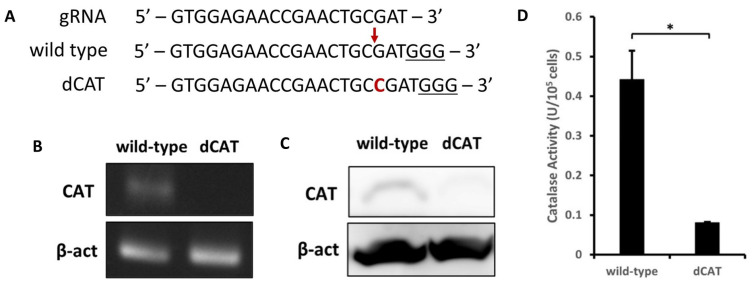
Generation of catalase-knockout PANC-1 cells. (**A**) Scheme of the sequence alignment of CRISPR/Cas9-induced insertion mutation. The underlined nucleotides indicate the PAM sequence, the nucleotide in red letter (**C**) indicates a 1-nt insertion (cytosine), and the red arrow indicates the DNA cleavage site. (**B**) The mRNA expression of catalase (CAT) (129 bp) was analyzed through reverse transcription on 2% agarose gel. β-actin (294 bp) was used as the housekeeping gene for normalization. (**C**) Western blotting analysis of catalase (66 kDa) in wild-type and dCAT cells. β-actin (44 kDa) served as the housekeeping gene. (**D**) The enzymatic activity of catalase in dCAT cells was lower than that found in wild-type cells due to the mutation (n = 3). Statistical significance was assessed using Student’s *t*-test. Each value is the mean ± standard deviations (SD), * *p* < 0.05.

We generated catalase-knockout PANC-1 (dCAT) cells to analyze whether modulating catalase in PANC-1 cells could enhance the radio-sensitizing effect of PAA-TiOx NPs to X-ray radiation on cells. CRISPR/Cas9 vector targeting exon 3 in the catalase gene resulted in a single nucleotide (1-nt) insertion in PANC-1 cells, establishing dCAT cells (Figure S2). The result was expected because previous studies reported that early exons are functional target regions for gene knockout using the CRISPR/Cas9 system because of the impact on protein function [31,32,33]. The nucleotide insertion possibly resulted from the non-homologous end joining (NHEJ) as the DNA repair system following the double-strand break caused by Cas9 nuclease [34,35] because a DNA template donor was absent in this study. Although 1-nt insertion is a common outcome [36,37], deletion typically occurs more than insertion when the nucleotide at position −4 from the protospacer adjacent motif (PAM) sequence is cytosine [38]. In this study, the nucleotide at position −4 upstream of the PAM sequence was also cytosine (C) (Figure 1A). However, the cleavage site was found at position −4 upstream of the PAM sequence, and guanine was present at the PAM-proximal end. These conditions cause a staggered 5′ end instead of a blunt end after DNA cleavage [37,39]. Staggered ends overhanging the PAM-distal end were reported to frequently result in an insertion [36,40] with an insert most likely identical to the nucleotide at the PAM-distal end [36,37,41]. Our study results align with previous studies as we observed a cytosine base as the insert, which is homologous to the nucleotide at position −4 from the PAM sequence (Figure 1A).

Next, we analyzed the effects of the mutation on the catalase expression and enzymatic activity. Following reverse transcription, the mRNA expression profile was visualized on 2% agarose gel. The catalase band (129 bp) density from dCAT cells was barely visible (Figure 1B), reflecting less transcription than wild-type cells. Densitometric analysis of the agarose gel bands indicated a 10-fold decrease in the expression compared with that of wild-type cells (*p* < 0.01) (Figure S3A). Both dCAT and wild-type cells were subjected to Western blotting to confirm whether this transcriptional change influences the protein level. A distinct band was detected at 66 kDa from wild-type cells, corresponding to the catalase protein (Figure 1C). On the other hand, a faint band was observed from dCAT cells with a 5-fold lower level of band intensity (*p* < 0.05) (Figure S3B), which indicates that transcriptional activity is linearly correlated with the protein level. To further ensure the catalase gene knockout, enzymatic activity was calculated from total protein by assessing the remaining H_2_O_2_ after reaction using 3,5-dichloro-2-hydroxy-benzenesulfonic acid (DHBS), 4-aminoantipyrine (AAP), and horseradish peroxidase (HRP) as a catalyst. The result showed that the mutation had significantly suppressed catalase activity by 81.7% (0.081 ± 0.001 U/10^5^ cells) from wild-type cells (0.442 ± 0.073 U/10^5^ cells) (*p* < 0.05) (Figure 1D). These findings suggest that the constructed CRISPR/Cas9 vector successfully induced a mutation on PANC-1 cells, obtaining dCAT cells with impaired catalase expression and enzymatic activity.

### 2.3. Catalase Knockout Reduces Cell Viability Following Treatment with PAA-TiOx NPs and X-Ray Radiation

**Figure 2:**
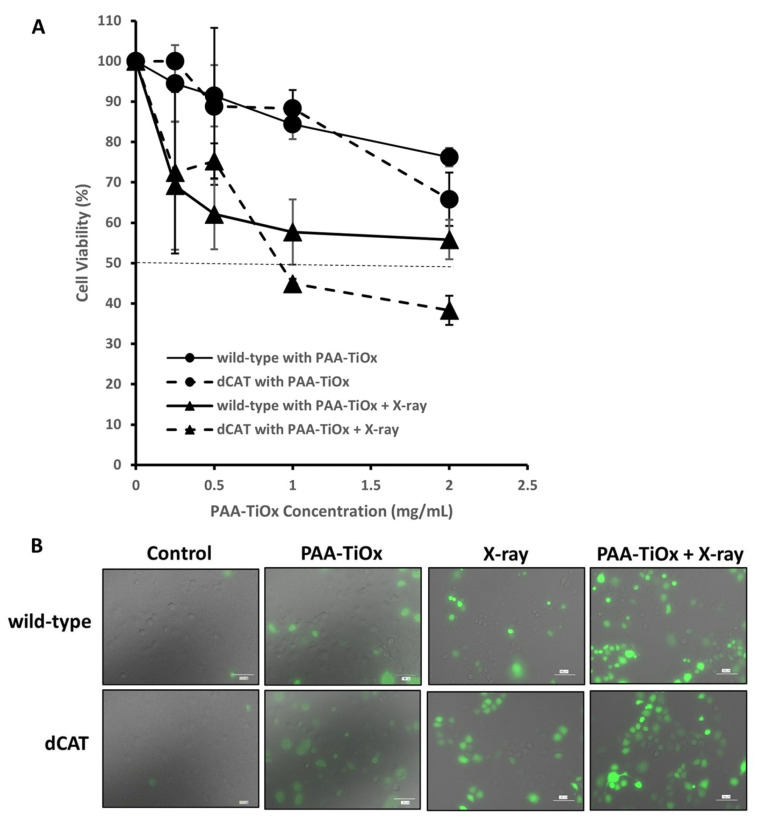
Cell viability assay. (**A**) Cytotoxicity of PAA-TiOx NPs with and without X-ray irradiation (5 Gy) on dCAT and wild-type cells. No significant difference was shown (n = 3). (**B**) Observation of total ROS generation after treatments on both cells with 1 mg/mL PAA-TiOx NPs. Green fluorescence indicates ROS generation.

A viability assay was conducted on dCAT and wild-type cells to assess the role of catalase in determining cellular responses to PAA-TiOx NPs and X-ray irradiation treatments. Cells were exposed to PAA-TiOx NPs at several concentrations for 1 h, which was followed by X-ray 5 Gy. Prior studies have established that the highest radio-sensitizing efficiency values were achieved when 1 mg/mL PAA-TiOx NPs were combined with X-ray 5 Gy on BxPC-3 cells [30]. In the present work, wild-type PANC-1 cells exhibited a concentration-dependent cytotoxicity effect from treatments of PAA-TiOx NPs alone and when combined with X-ray radiation (Figure 2A). The observed results differ from those reported in the previous study, which found that 1 h incubation of PAA-TiOx NPs showed no cytotoxicity to BxPC-3 cells [30]. This discrepancy could have been due to differences in the cell lines. BxPC-3 cells have a higher natural expression of AKT (protein kinase-B) than PANC-1 cells [42]. In hallmarks of cancer, AKT promotes oncogenesis by inhibiting the apoptosis that leads to cell proliferation [43]. Although PAA-TiOx NPs combined with X-ray radiation can inhibit AKT and induce apoptosis in pancreatic cancer [26,44], cells with high AKT expression may exhibit lower levels of susceptibility to treatment with PAA-TiOx NPs.

Following the PAA-TiOx NPs treatment alone, dCAT cells showed comparable viability to wild-type cells (Figure 2A). Meanwhile, in combination with X-ray radiation, 1 mg/mL PAA-TiOx NPs resulted in a dCAT cells viability of 44.9%, a 1.3-fold decrease from the viability of wild-type cells (57.7%). A consistent response was shown with 2 mg/mL PAA-TiOx NPs. dCAT cell viability (38.3%) demonstrated a 1.5-fold decrease from levels observed in wild-type cells (55.8%). Since dCAT cells had impaired catalase expression and activity, exposure to the combination treatment could have led to an accumulation of H_2_O_2_ because its conversion to water and oxygen was inhibited, as shown in the total ROS observation (Figure 2B). Hence, the ability of cells to neutralize total ROS was limited. This redox homeostasis disruption in dCAT cells could influence both cell viability and ROS production. However, the differences between both types of cells did not reach statistical significance under the experimental conditions. 

### 2.4. Catalase Knockout Improves PANC-1 Cells Susceptibility to PAA-TiOx NPs and X-Ray Radiation in Hypoxia

Hypoxia is a common characteristic in solid tumors, including pancreatic cancer, because extensive fibrosis in solid tumors inhibits proper vascular development and results in hypovascularization [45,46]. In cancer treatments, the low-oxygen conditions in hypoxic tumors result in undesirable outcomes, such as radiation resistance [12,13,47] and metastasis of cancer cells [48,49]. Compared with the rates seen in normoxia, hypoxia causes less permanent DNA damage and ROS generation induced by ionizing radiation [47]. Therefore, a higher radiation dose is often required under hypoxia in order to obtain the same level of radiation effect as that under normoxia [12,47]. Herein, we analyzed whether catalase knockout modulates the synergistic effect of PAA-TiOx NPs and X-ray radiation in hypoxic conditions.

dCAT and wild-type cells were incubated under 1% oxygen for 24 h to stimulate hypoxic conditions. A cellular hypoxic state was confirmed by assessing the expression of hypoxia-inducible factor 1-alpha (HIF1-α) transcription factor. HIF1-α is a subunit of heterodimer HIF1, which facilitates cell adaptation under low-oxygen conditions that are identified by the stabilized expression of HIF1-α in hypoxia [48,50]. Normoxia, a condition characterized by 18–19% oxygen, was used as a control group. The present study demonstrated that HIF1-α was stabilized at a level of 1% oxygen for 24 h for both dCAT and wild-type cells (Figure S4). These results agree with those found in a study by Zhao et al., which mimicked hypoxia in PANC-1 cells via 24 h of incubation under 1% oxygen [51]. In wild-type cells, HIF1-α mRNA and protein were expressed even under normoxia. Figure S4A shows a prominent band at 424 bp, which indicates HIF1-α mRNA expression under both normoxia and hypoxia. HIF1-α protein was also detected under normoxia, although it appeared as a faint band (93 kDa) (Figure S4B). This result agrees with another study, which revealed that the HIF1-α protein is expressed even under non-hypoxic conditions in pancreatic cancer cells, including PANC-1 cells [52]. However, we observed that the protein expression was not constitutive because the HIF1-α protein was more stabilized under hypoxia with a 14.5-fold increase in protein expression (Figure S4C). This result demonstrated a general cellular response to hypoxia. Although the HIF1-α protein is continuously synthesized, proteasomal degradation could occur in normoxia because oxygen activates post-translational hydroxylation involving prolyl hydroxylase enzymes. Under hypoxia, therefore, hydroxylase enzyme activity is hindered, which leads to a stable accumulation of HIF1-α [53,54,55]. The variable results, however, could have been caused by variations in the experimental conditions.

On the other hand, dCAT cells showed no apparent HIF1-α mRNA expression under normoxia (Figure S4A). This result could have been caused by the disruption of redox status in dCAT cells, which increases the activity of glutathione (GSH) because it is a burden to maintain redox homeostasis in the catalase deficiency condition, leading to the destabilization of HIF1-α [56]. The decreased mRNA expression was also reflected at the protein level, with a 2-fold decrease in protein expression compared with wild-type cells (Figure S4B,C). In hypoxia, HIF1-α expression in dCAT cells exhibited a typical manner in responses to low-oxygen conditions, with a 12.8-fold increase in protein expression. Finally, HIF1-α stabilization under hypoxia was confirmed in dCAT and wild-type cells.

Next, the established hypoxic conditions were employed to examine catalase activity from dCAT and wild-type cells in order to learn whether reduced oxygen levels affect the enzymatic activity in both types of cells. Catalase activity in wild-type cells was increased under hypoxia by as much as 47.7% (0.644 ± 0.245 U/10^5^ cells) or a 1.5-fold increase compared with that under normoxia (0.436 ± 0.031 U/10^5^ cells) (Figure S4D). The escalated activity under hypoxia could have resulted from adenosine monophosphate-activated protein kinase (AMPK) pathway activation, which is known to boost transcription factor FoxO1 and induce catalase expression and activity [57,58]. Meanwhile, due to the confirmed catalase knockout, consistent catalase activity from dCAT cells was observed under both normoxia (0.193 ± 0.003 U/105 cells) and hypoxia (0.193 ± 0.017 U/105 cells).

**Figure 3:**
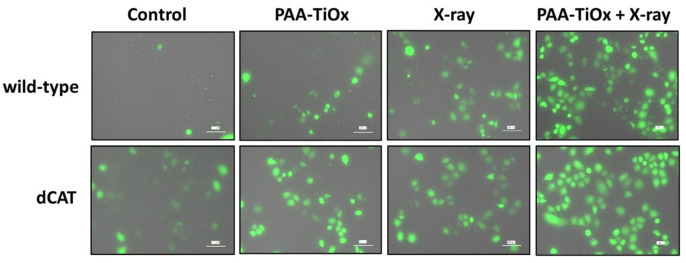
Observation of total ROS in dCAT and wild-type cells under hypoxia (1% oxygen). Catalase knockout induced a greater generation of ROS in PANC-1 cells. Green fluorescence indicates ROS generation. The scale bar indicates 100 µm.

The catalase activity profile from both cells under normoxia and hypoxia may be attributed to the observed total ROS generation in Figure 2B and Figure 3, which influences the redox balance in the cells. As shown in Figure 3, total ROS was observed more in the control group of dCAT cells than in wild-type cells. This finding may reflect the naturally increased endogenous H_2_O_2_ and radical ROS under hypoxia [57,58], and the incapability of dCAT cells to maintain a redox status due to the catalase knockout. A distinct increase in total ROS generation was observed in dCAT cells after treatment with PAA-TiOx NPs alone compared with that in wild-type cells. Nonetheless, comparable ROS production was observed after treatment with only X-ray irradiation. These results indicate that modulating catalase in PANC-1 cells under hypoxic conditions induces a more potent effect of PAA-TiOx NPs on cells by increasing ROS generation. Generally, a more pronounced effect was shown by combination treatment as ROS production was elevated. However, the difference between dCAT and wild-type cells was comparable. The rationale is that the observed ROS production could have reached the maximum threshold under the experimental conditions, beyond which no clear difference was observed.

**Figure 4:**
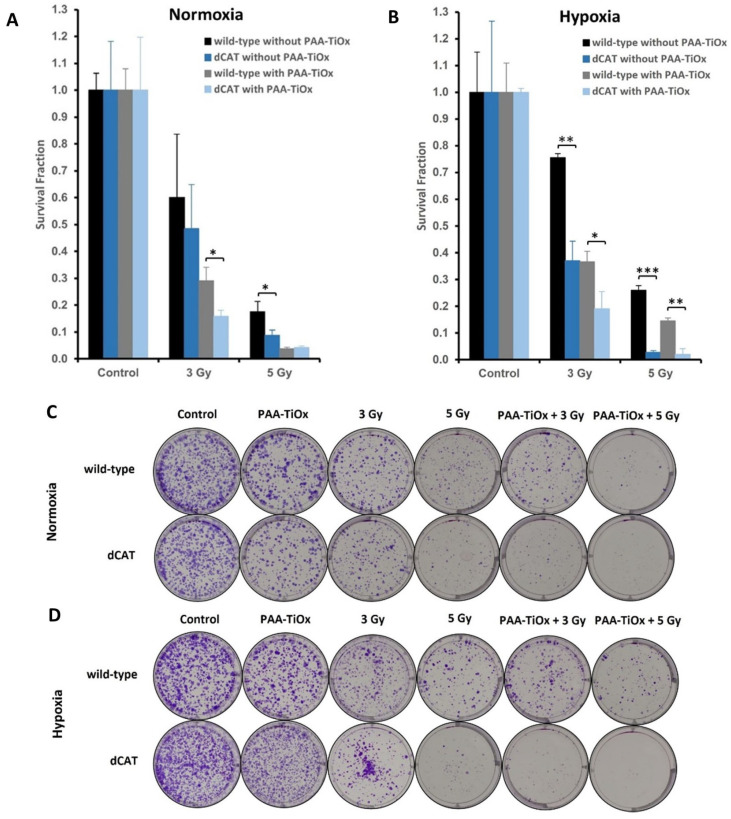
Colony formation results of dCAT and wild-type cells. (**A**) Survival fraction under normoxia. (**B**) Survival fraction under hypoxia. (**C**,**D**) Representative plates of stained colonies (1500 cells) under (**C**) normoxia and (**D**) hypoxia. Purple color indicates stained colonies. Statistical significance was assessed using Student’s *t*-test. Each value is the mean ± standard deviations (SD) (n = 3), * *p* < 0.05, ** *p* < 0.01, *** *p* < 0.001.

The generation of total ROS in both types of cells was then confirmed for its contribution to survival ability. Following treatments under normoxia (Figure 4A) and hypoxia (Figure 4B), dCAT cells generally exhibited a lower ability to survive compared with that of wild-type cells. The differences were significant under hypoxic conditions, which aligned with the total ROS production. However, in normoxia, the survival fraction (SF) value of wild-type and dCAT cells showed no distinction with X-ray 5 Gy. These results are inconsistent with the results of the cell viability assay, depicted in Figure 2A, with the same dose of PAA-TiOx NPs and X-ray radiation. The rationale for the inconsistency may be due to different measurement parameters and assay sensitivity. Cell viability assay measures metabolic activity, and slight reductions in the viability may be detected. On the other hand, the colony formation assay measures the ability of the cells to survive the treatments and form colonies. Only those cells that can proliferate and form visible colonies are counted. Therefore, cells may be metabolically active in the cell viability assay but unable to form colonies.

The results from the colony formation assay suggest that an increase in the ROS in dCAT cells contributes to an increase in cell death. A significant difference in the SF value was observed between dCAT and wild-type cells following the combination treatment of PAA-TiOx NPs and X-ray 5 Gy under hypoxia. dCAT cells exhibited an SF value of 0.021 ± 0.021, while the survival of wild-type cells was more significant with an SF of 0.146 ± 0.010 (*p* < 0.01). A similar pattern was also shown with a smaller X-ray radiation dose (3 Gy). The survival of dCAT cells was typically reduced under hypoxia than under normoxia following both single and combination treatments. On the contrary, wild-type cells showed greater persistence despite treatments under hypoxia. The underlying cause of this discrepancy could be that the stabilized HIF1-α in p53-mutated pancreatic cancer cells induces cell protection from apoptosis. The protection mechanism is likely a result of the stabilization of the mitochondrial transmembrane potential through enhanced glucose uptake and anaerobic metabolism [59,60]. PANC-1 cells were used in this study, and were reported to have a mutation in the TP53 tumor suppressor gene [61]. Another explanation could be solely because of the radiotherapy resistance caused by oxygen deficiency [12,13,47]. In addition, the increased catalase activity in wild-type cells under hypoxic conditions (Figure S4D) could also contribute to the ability of wild-type cells to endure such treatments. Although exogenous H_2_O_2_ from PAA-TiOx NPs could decrease catalase activity [57,58], the decline in activity is likely adequate to maintain H_2_O_2_ and total ROS at moderate levels; thus, wild-type cells survive and proliferate to a greater extent.

Moreover, an intriguing outcome was observed under hypoxic conditions. The combination treatment of PAA-TiOx NPs and X-ray 3 Gy in dCAT cells resulted in an SF that was similar to that observed in wild-type cells treated with the same combination but at a higher radiation dose (5 Gy). These results suggest that PANC-1 cells demonstrate a higher susceptibility to PAA-TiOx NPs combined with X-ray radiation when under an impaired catalase state.

Under normoxia, PANC-1 cells treated with PAA-TiOx NPs, X-ray radiation, and under reduced catalase demonstrated reduced survival. Under hypoxia, with a natural increase in ROS and the same conditions as normoxia, oxidative stress could be exacerbated in dCAT cells, which would lead to a disruption of redox homeostasis. dCAT cells were unable to accommodate the stress. Consequently, the cells barely survived. Overall, these results suggest that (1) inhibition of catalase enhances the effect of PAA-TiOx NPs when combined with X-ray radiation in PANC-1 cells; (2) ROS-induced damages in PANC-1 cells under catalase deficiency are not limited to one oxygen condition. The effect is even more significant under hypoxia than that of PANC-1 cells under the normal state of oxygen. Notably, the results indicate the importance of catalase in regulating the therapeutic effect of PAA-TiOx NPs and X-ray radiation in pancreatic cancer cells.

There are some limitations to our study. Our dataset currently lacks apoptosis analysis. To overcome this limitation, we are investigating apoptosis-related pathways and the roles they could play in the observed effects to gain further insights into the mechanism of action. Employing the CRISPR/Cas9 system in clinical settings to obtain the impaired catalase state also faces potential limitations, such as delivery method into cells and target specification [62]. Moreover, catalase inhibition may be detrimental for normal cells because of induced oxidative stress, although inhibition in tumor cells benefits the host organisms [63]. Hence, to address these challenges, applying non-toxic catalase inhibitors that affect less normal cells should be a feasible alternative in clinical settings to modulate catalase activity and improve the combination of PAA-TiOx NPs and X-ray irradiation efficacy.

An example of a catalase inhibitor for cancer cells is L-ascorbic acid (ascorbate). It was reported to reduce catalase protein expression in human pancreatic cell lines, MIAPaCa-2 and PANC-1, with or without 5 Gy irradiation. Interestingly, catalase inhibition by ascorbate was not observed in non-tumorigenic H6c7 and FhsInt cell lines [64], and ascorbate also has the potential to protect normal cells from radiation-induced damage [65]. Other catalase inhibitors are salicylic acid and anthocyanidins. Inactivation of tumor cell protective catalase by salicylic acid is direct through the transformation of compound I of catalase into inactive compound II. On the other hand, anthocyanidins inhibit catalase indirectly, which is mediated by the singlet oxygen-dependent inactivation of catalase. The different biochemical mechanisms were reported to make a synergistic combination of salicylic acid and anthocyanidins to induce apoptosis in MKN-45, a human gastric cancer cell line [66]. A synthetic salicylic acid derivative, acetyl salicylic acid (aspirin), is commonly used to reduce inflammation. Some natural salicylic acid derivatives also possess promising pharmacological effects [67]. Cyanidins, an anthocyanidins variety, are distributed in plants and cause the reddish-purple hue of some vegetables and fruits [68]. Cyanidins isolated from banana floral bract were reported for various therapeutic activities, such as anti-inflammatory and anti-proliferative. It also shows oral safety in rats with a No Observed Adverse Effect Level (NOAEL) at 30 mg/kg/day [69]. Although these catalase inhibitors possess additional pharmacological benefits, further research on these catalase inhibitors combined with PAA-TiOx NPs and X-ray radiation is necessary to ensure the therapeutic combination doses and side effects, and validate the in vitro findings to be translated to in vivo and clinical studies.

## 3. Materials and Methods

### 3.1. Synthesis of PAA-TiOx NPs

PAA-TiOx NPs were synthesized according to a previously established method [25]. Titanium dioxide (TiO_2_) NPs (STS-01, Ishihara Sangyo Kaisha, Osaka, Japan) were surface-modified with PAA (MW 5000, Wako Pure Chemical Industries Ltd., Osaka, Japan) via a hydrothermal process. PAA-TiOx NPs were then collected by centrifugation at 5000× *g* for 10 min and immersed in a 6% H_2_O_2_ solution. Ultrafiltration was conducted 5 times (5000× *g*, 30 min) to wash away the remaining H_2_O_2_ molecules using a centrifugal filter (Amicon 10 kDa MWCO, Millipore, Darmstadt, Germany). After further centrifugation (10,000 rpm, 30 min), the residue was dispersed in 12 mL distilled water, vortexed, and sonicated for 5 min before storage at 4 °C with avoidance of light. Particle sizes and zeta potentials were determined using a Zetasizer Nano ZS (Malvern Instrument Ltd., Malvern, UK).

### 3.2. Cell Culture

The human pancreatic cancer cell line, PANC-1, was obtained from the JCRB Cell Bank (Osaka, Japan) and cultured in a complete medium consisting of Dulbecco’s modified Eagle medium (DMEM) High Glucose (Nacalai Tesque, Inc., Kyoto, Japan) supplemented with 10% fetal bovine serum (FBS) and 1% antibiotic solution (100 U/mL penicillin, 100 µg/mL streptomycin, and 0.25 µg/mL amphotericin B). Cells were maintained at 37 °C in a 5% CO_2_ incubator, washed with Dulbecco’s phosphate-buffered saline (D-PBS), and harvested using trypsin-EDTA.

### 3.3. Normoxia and Hypoxia Conditions

Normoxia was defined as a culture condition at standard incubator settings at 18–19% oxygen levels, while hypoxia was maintained at 1% oxygen. After 24 h incubation, cells were prepared for hypoxia using a Bionix Hypoxic Culture Kit (Sugiyamagen, Tokyo, Japan) and then incubated for another 24 h. Treatments under hypoxic conditions were conducted in a growth chamber flushed with Nitrogen gas to achieve the desired oxygen concentration. An anaerobic jar and bags (Mitsubishi Gas Chemical Company, Inc., Tokyo, Japan) were also used in treatments under hypoxia.

### 3.4. X-Ray Radiation

Irradiation was performed using an MX-160Labo (Medi-X-Tech Co., Ltd., Chiba, Japan) with an X-ray tube at a voltage and current of 160 kV and 3.0 mA, respectively. The distance of the treatment table to the X-ray source was 300 mm, with a dose rate of 0.6 Gy/min.

### 3.5. Construction of CRISPR/Cas9 Vector

Plasmids targeting catalase knockout were based on a CRISPR/Cas9 system utilizing the H1 promoter and CMV promoter to drive the expression of guide RNA (gRNA) and Cas9 endonuclease, respectively. gRNA was designed using the CHOPCHOP (https://chopchop.cbu.uib.no, accessed on 16 November 2022) web tool with an NGG-PAM sequence [70]. The synthesis of gRNA was performed at 95 °C for 5 min with 18 µL of annealing buffer (10 mM Tris pH 8, 50 mM NaCl, 1 mM EDTA) and 1 µL each of forward and reverse primer (10 mM). Additional bases were added to the 5′-end of the primers to complement the overhangs on the digested plasmid. The plasmid was digested with *Sap*I using CutSmart Buffer (New England Biolabs Inc., Ipswich, MA, USA) overnight at 37 °C, followed by incubation at 65 °C for 40 min. Subsequently, a linearized plasmid was used to ligate the synthesized gRNA using the Ligation High procedure (Toyobo Co. Ltd., Osaka, Japan) at 16 °C overnight. The established plasmid vector was confirmed by sequence analysis using a BigDye Terminator v3.1 Cycle Sequencing Kit (Applied Biosystems, Foster City, CA, USA). The primers used for gRNA synthesis and plasmid vector confirmation are listed in Table S2. All DNA concentrations were determined using a NanoDrop One spectrophotometer (Thermo Fischer Scientific, Waltham, MA, USA).

### 3.6. Cloning and Isolation of a Vector

The prepared plasmid vector was transformed to competent *Escherichia coli* NovaBlue for cloning purposes. One µL of plasmid vector (1 ng/µL) was added to 50 µL of competent cells, which was followed by incubation on ice for 40 min and heat shock at 42 °C for 40 s. Further incubation on ice for 5 min was immediately conducted before spreading the mixture on LB agar plates (10 g/L tryptone, 5 g/L yeast extract, 5 g/L NaCl, 15 g/L agar) containing 100 µg/mL ampicillin. The plates were inverted and incubated overnight at 37 °C. Single colonies were individually picked and grown in LB broth media (overnight, 37 °C, 200 rpm) for plasmid vector purification using Wizard Plus SV Minipreps (Promega, Madison, WI, USA) according to the manufacturer’s instructions. A confirmed, purified plasmid vector was then employed for cell transfection.

### 3.7. Cell Transfection and Antibiotic Selection

PANC-1 cells (2 × 10^5^ cells/well) were seeded into 6-well plates in 2 mL of media and cultured for 24 h. Prior to the transfection, the medium was replaced with 1 mL of antibiotic-free DMEM. CRISPR/Cas9 vector transfection was carried out using a Lipofectamine 3000 (Invitrogen, Carlsbad, CA, USA) according to the manufacturer’s instructions. The concentration of the plasmid vector was 2.5 µg with 3.75 µL of transfection reagent. The cells were subjected to a 24 h recovery period before adding 1 µg/mL puromycin (Invivogen, New Territories, China) in the medium for 5 days. The limiting dilution method was performed in 96-well plates (0.8 cell/well) to isolate single clones. Selected clones were grown and expanded to allow confirmation of the catalase knockout and further analyses.

### 3.8. Detection of mRNA Expression

dCAT and wild-type PANC-1 cells (1 × 10^6^ cells/well) were seeded into 6-well plates for 24 h. Total RNA was isolated using NucleoSpin RNA (Macherey-Nagel, Duren, Germany), which was subsequently used (0.25 µg) for complementary DNA (cDNA) synthesis using a ReverTraAce qPCR RT Master Mix with a gDNA Remover (Toyobo, Osaka, Japan) according to the manufacturer’s instructions. A no-reverse transcription mixture was used as a control for the experiment. Amplification of cDNA for expression studies was conducted for 35 cycles of denaturation at 98 °C for 10 s, annealing at 60 °C for 5 s, and extension at 68 °C for 3 s. Primers used for the amplification were as follows: CAT (129 bp) forward primer: 5′—AGGGGCCTTTGGCTACTTTGAG -3′, CAT reverse primer: 5′—GAACCCGATTCTCCAGCAACAG—3′; HIF1-α (424 bp) forward primer: 5′—CCTAACGTGTTATCTGTCGC—3′, HIF1-α reverse primer: 5′—GTCAGCTGTGGTAATCCACT—3′; β-actin (294 bp) forward primer: 5′—TCACCCACACTGTGCCCATCTACGA—3′, β-actin reverse primer: 5′—AGCGGAACCGCTCATTGCCAATGG—3′. Amplicons were separated on 2% agarose gel in TAE buffer, and densitometric analysis was performed using ImageJ software (version 1.54, National Institutes of Health, Bethesda, MD, USA). Relative band intensities were calculated by normalizing the CAT to β-actin gene bands as the housekeeping gene.

### 3.9. Western Blotting

Cells (0.5–1 × 10^6^ cells/well) were seeded into 6-well plates for 24 h and treated as necessary. Cells were washed twice with D-PBS (Nacalai Tesque, Inc., Kyoto, Japan) and lysed with RIPA buffer containing protease inhibitor cocktail (Nacalai Tesque, Inc., Kyoto, Japan) according to the manufacturer’s instructions. Cell lysates were then subjected to a bicinchoninic acid (BCA) assay (Nacalai Tesque, Inc., Kyoto, Japan) to determine the protein concentration. An equivalent protein (36 µg) was loaded into a 15% precast SDS-PAGE gel and separated at 20 mA for approximately 70 min. Both protein marker Precision Plus Protein Dual Color Standards (Biorad, Hercules, CA, USA) and a Magic Mark XP Western Protein Standard (Invitrogen, Carlsbad, CA, USA) were loaded into each gel for SDS-PAGE and Western blotting markers, respectively. SDS-PAGE gel was then transferred to a polyvinylidene difluoride (PVDF) membrane from iBlot 2 Transfer Stacks (Invitrogen, Carlsbad, CA, USA) using an iBlot 2 Dry Blotting System (Invitrogen, Carlsbad, CA, USA). The transfer was performed at 20 V for 1 min, 23 V for 4 min, and 25 V for 3 min, according to the manufacturer’s instructions.

A PVDF membrane was blocked using Blocking One (Nacalai Tesque, Inc., Kyoto, Japan) at 4 °C overnight and then probed with primary antibody at room temperature for 1 h with gentle agitation on a rocker shaker. Afterward, the membrane was washed with TBS-Tween (0.1% Tween 20) three times (5 min each time). The membrane was subsequently incubated with a secondary antibody for 1 h and then washed with TBS-Tween as the previous step. Primary and secondary antibodies were diluted in Blocking One: TBS-Tween (1:5) solution. The antibodies used in this study were as follows: anti-catalase antibody (1:600, ab16731, Abcam, Cambridge, UK); anti-HIF-1α (1:600, ab228649, Abcam, Cambridge, UK); anti-beta actin (1:3000, ab8227, Abcam, Cambridge, UK); and HRP-conjugated Rabbit Anti-Goat IgG(H+L) (1:3000, Proteintech, Rosemont, IL, USA). Chemiluminescence detection was conducted using an ImmunoStar Zeta (Wako Pure Chemical Industries Ltd., Osaka, Japan). The density of protein expression bands was analyzed using ImageJ software (version 1.54, National Institutes of Health, Bethesda, MD, USA) with normalization to β-actin protein bands.

### 3.10. Catalase Activity

Cells (2 × 10^5^ cells/mL) were collected and lysed using RIPA buffer. Centrifugation at 10,000× *g* for 15 min at 4 °C was performed to collect the cell lysates, and enzyme activity was determined using OxiSelect Catalase Activity Assay Kit (Cell Biolabs, Inc., San Diego, CA, USA) according to the manufacturer’s instructions. Briefly, cell lysates were incubated in H_2_O_2_ solution (12 mM) for 1 min, and the reaction was quenched with sodium azide. The remaining H_2_O_2_ was subsequently reacted for 1 h with DHBS, AAP, and HRP as a catalyst. The quinoneimine dye, as the reaction product, was measured at 520 nm, which correlated with the remaining H_2_O_2_ in the mixture. Catalase activity was quantified by comparing the absorbance with a standard curve prepared using a known catalase enzyme concentration. Results were normalized to cell density and expressed as units per one hundred thousand cells (U/10^5^ cells).

### 3.11. Cell Viability Assay

Cells (1 × 10^4^ cells/well) were grown in 96-well plates for 24 h and incubated for 1 h in PAA-TiOx NPs (0.25, 0.5, 1, and 2 mg/mL) diluted in D-PBS. After applying PAA-TiOx NPs, treatment groups were divided into with and without X-ray radiation at a dose of 5 Gy. The control group for each wild-type and dCAT cell was incubated without the addition of PAA-TiOx NPs and X-ray irradiation. Afterward, cells were incubated in a medium for a further 24 h. Cell Counting Kit-8 (CCK-8; Dojindo, Kumamoto, Japan) reagent (10%) was added into each well, followed by incubation for 2 h at 37 °C. Absorbance was observed at 450 nm with blank subtraction (medium only). Cell viability was normalized to the absorbance of the control group and expressed in percentages.

### 3.12. Observation of Total ROS

Cells (1 × 10^4^ cells/well) were seeded into a 96-well black plate with a clear bottom under normoxia and hypoxia. Cell treatments were performed with PAA-TiOx NPs (1 mg/mL), X-ray 5 Gy, or a combination of PAA-TiOx NPs and X-ray irradiation. Total ROS production was observed using a Total ROS Detection Kit (Dojindo, Kumamoto, Japan) according to the manufacturer’s instructions. Briefly, treated cells were washed with Hank’s Balanced Salt Solution (HBSS) and cultured in the medium for 30 min at 37 °C. After further washing with HBSS, 2,7-dichlorofluorescein diacetate (DCFH-DA) solution was added to the cells for 30 min. Cells were washed with HBSS, and total ROS was observed in HBSS using a fluorescence microscope.

### 3.13. Colony Formation Assay

Cells (1 × 10^5^ cells/well) were seeded into 6-well plates under normoxia and hypoxia. Treated cells were trypsinized and counted using a Moxi Z Cell Counter (ORFLO, El Cajon, CA, USA). Cells were re-seeded in 6-well plates at various densities ranging from 300 to 5000 cells/well for 12–14 days. Colony fixation was performed with methanol for 20 min and staining using 0.5% crystal violet in 25% methanol for 5 min. Plating efficiency (PE) was calculated as the ratios of colonies formed to the number of cells plated initially using Equation (1). The survival fraction (SF) was calculated using Equation (2) as the ratio of PE from the treated group to PE from the control group.

Equation (1) was used for the PE calculation.

(1) $$PE=\frac{\mathrm{Number}\;\mathrm{of}\;\mathrm{colonies}\;\mathrm{formed}}{\mathrm{Number}\;\mathrm{of}\;\mathrm{cells}\;\mathrm{plated}}$$

Equation (2) was used for the SF calculation.

(2) $$SF=\frac{\mathrm{PE}\;\mathrm{of}\;\mathrm{treated}\;\mathrm{sample}\;\mathrm{group}}{\mathrm{PE}\;\mathrm{of}\;\mathrm{control}\;\mathrm{group}}$$

### 3.14. Statistical Analysis

All data are expressed as the mean ± SD of at least two independent replicates. Differences between the cell groups were analyzed using a Student’s *t*-test. A *p* < 0.05 was considered as a significant difference.

## 4. Conclusions

Reduced catalase expression and activity in PANC-1 cells improved the synergistic effects of PAA-TiOx NPs and X-ray irradiation on the cells. The impaired catalase state in the cells increased their susceptibility to combination treatment by enhancing ROS production, which disrupted the redox balance and ultimately induced cell death. It is noteworthy that catalase inhibition is a promising strategy to advance the combination treatment of PAA-TiOx NPs and X-ray radiation in pancreatic cancer cells. However, to translate these in vitro findings to in vivo or clinical settings, the application of catalase inhibitors instead of the CRISPR/Cas9 system is a feasible approach to obtain the catalase inhibition in future studies.

## Data Availability

The original contributions presented in this study are included in the article/Supplementary Materials. Further inquiries can be directed to the corresponding author.

## Supplementary Materials

The following supporting information can be downloaded at: www.mdpi.com/article/10.3390/molecules30030629/s1, Figure S1: Characterization of PAA-TiOx NPs using dynamic light scattering; Figure S2: Generation of dCAT cells; Figure S3: Densitometric analyses of catalase expression; Figure S4: Analyses of HIF1α expression and catalase activity in normoxia and hypoxia. Table S1: Results of PAA-TiOx NPs characterization on size and zeta potential by using dynamic light scattering. Table S2: Primers used for gRNA synthesis and plasmid vector confirmation.
