# Supplementary material for: Catalase-Knockout Complements the Radio-Sensitization Effect of Titanium Peroxide Nanoparticles on Pancreatic Cancer Cells
Source: Molecules. 2025 Jan 31;30(3):629. doi: 10.3390/molecules30030629 (PMC11820024; doi:10.3390/molecules30030629)
Supplement: Supplementary file 1 [file molecules-30-00629-s001.zip › molecules-3425861-supplementary.pdf]

molecules

MDPI

Supplementary Materials

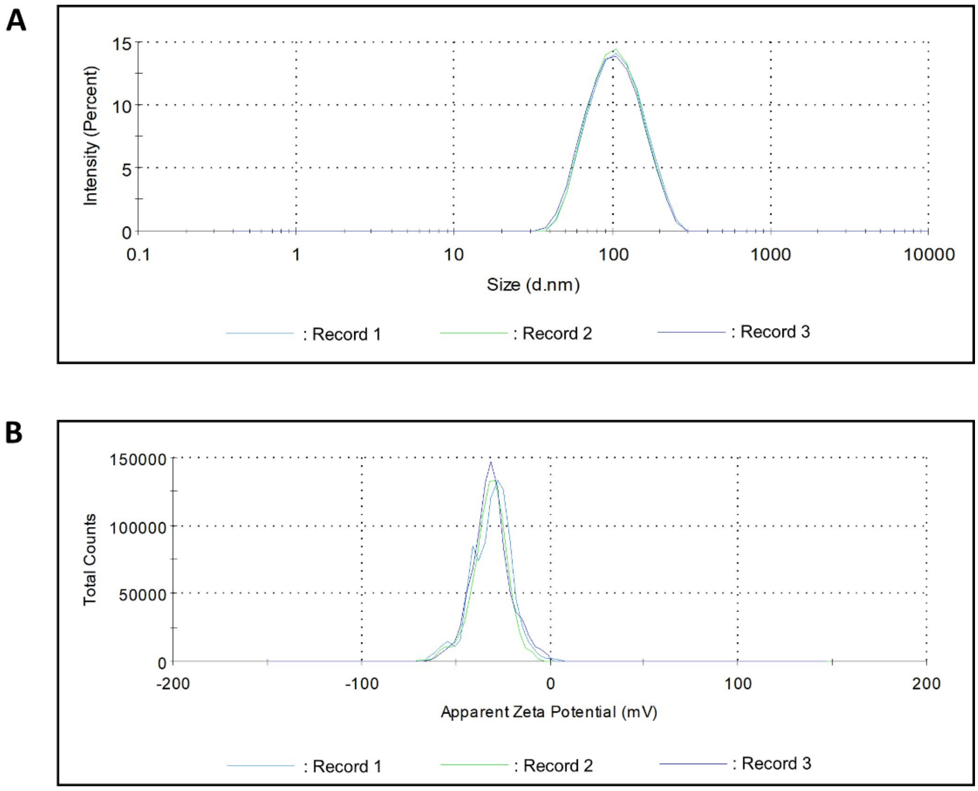

Figure S1. Characterization of titanium peroxide nanoparticles (PAA-TiOx NPs) using dynamic light scattering. (**A**) Size distribution with hydrodynamic diameter was found to be 94.3 ± 1.2 nm. (**B**) Zeta potential distribution with average number of -31.3 ± 0.4 mV.

Table S1. Results of PAA-TiOx NPs characterization on size and zeta potential by using dynamic light scattering.

	Size (d.nm)	PdI	Zeta Potential (mV)
Record 1	95.38	0.15	-30.9
Record 2	94.61	0.14	-31.9
Record 3	93.01	0.15	-31.1
Average	94.3 ± 1.2	0.15	-31.3 ± 0.4

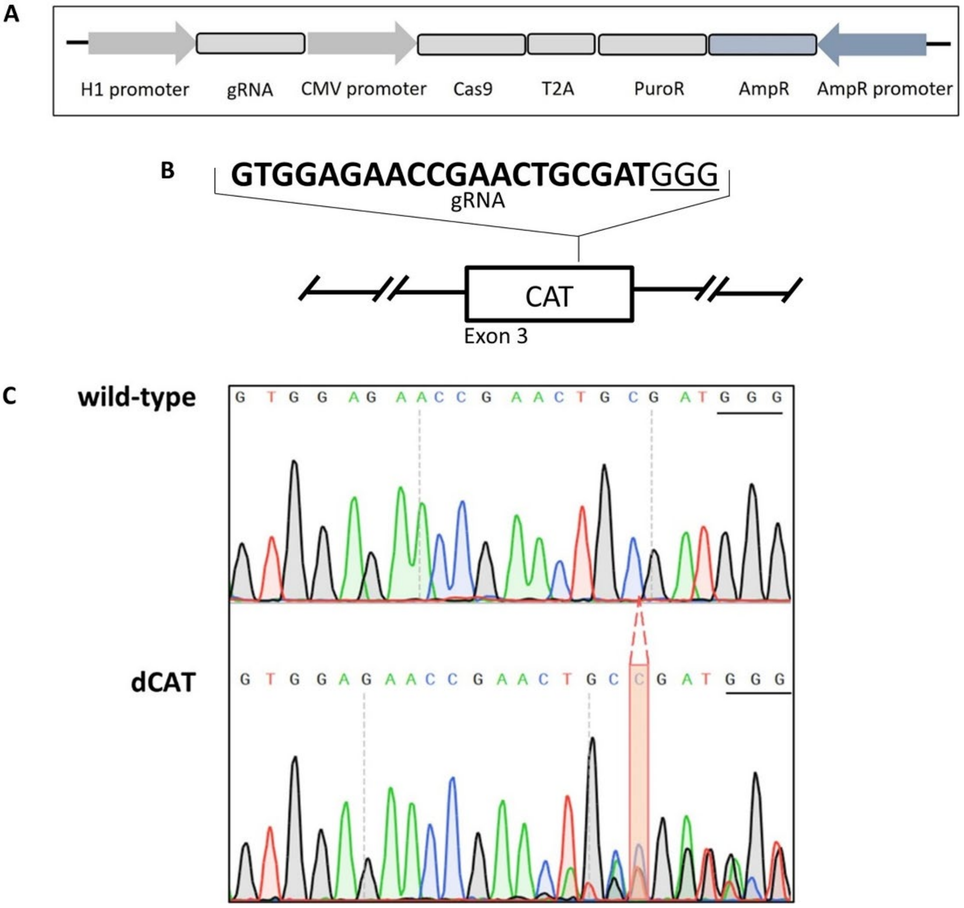

Figure S2. Generation of catalase-knockout PANC-1 (dCAT) cells. (**A**) Construction of CRISPR/Cas9 vector for catalase knockout in this study. (**B**) Target position of guide RNA (gRNA) used for catalase knockout and relative position in the human catalase gene (Gene ID: 847). Bold nucleotides indicate gRNA, and underlined nucleotides indicate protospacer adjacent motif (PAM) sequences. (**C**) Chromatogram of aligned wild-type and dCAT cells sequences. A single nucleotide insertion (cytosine) was observed at position -4 upstream from the PAM sequence. Underlined nucleotides indicate the PAM sequence.

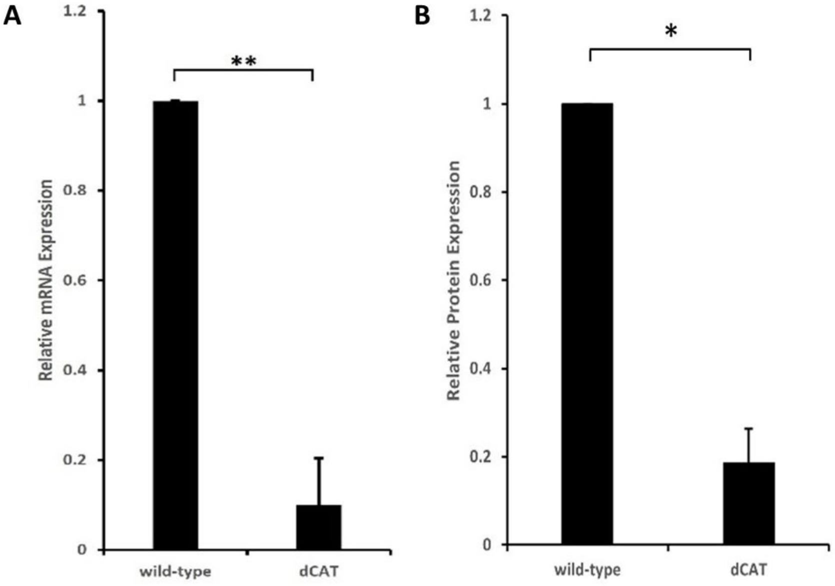

Figure S3. Densitometric analysis using an ImageJ of (**A**) mRNA expression of catalase visualized on agarose gel, following reverse transcription and (**B**) Western Blotting of catalase protein on wild-type and dCAT cells. Relative expression was quantified by

normalizing the band intensity to β-actin. Statistical significance was assessed using a Student's *t*-test. Each value is reported as the mean ± standard deviations (SD) (n=2), * $p < 0.05$, ** $p < 0.01$.

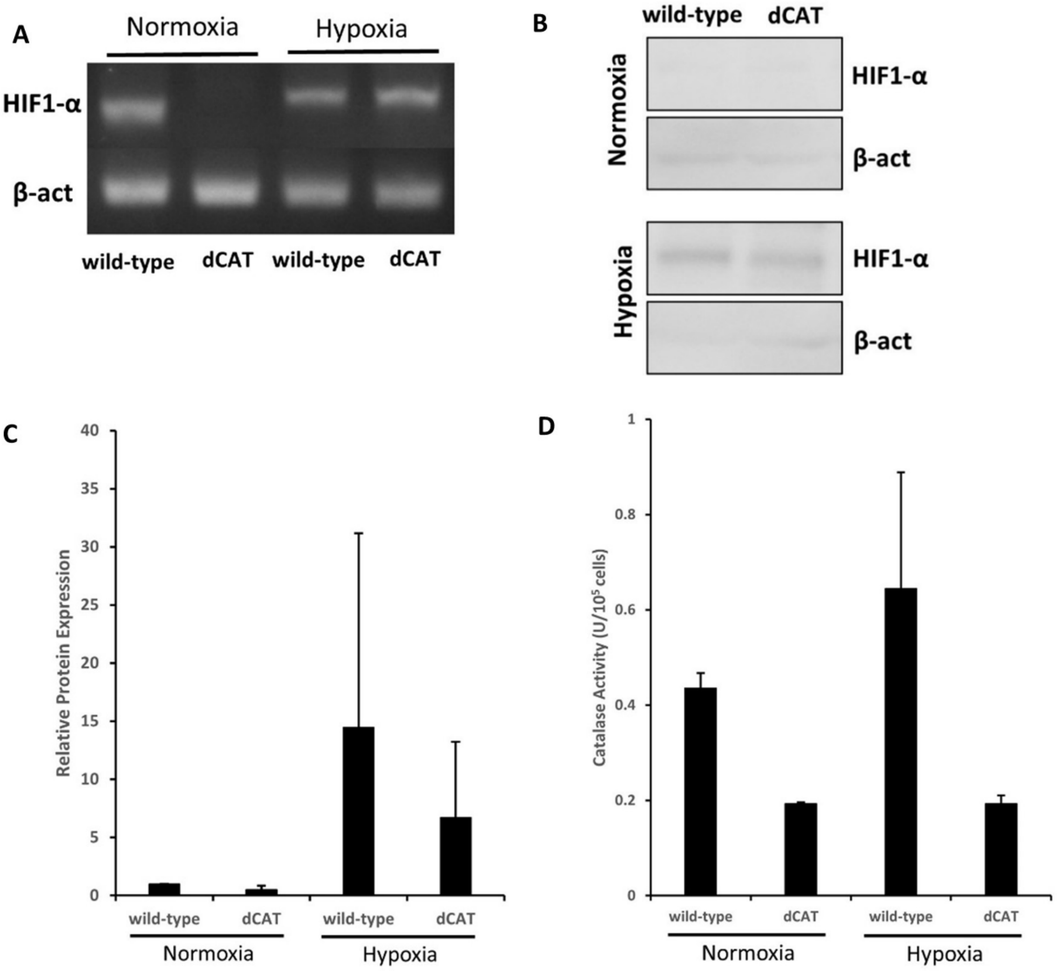

Figure S4. Analysis of HIF1-α expression and catalase activity in normoxia and hypoxia. (**A**) mRNA expression of HIF1-α (424 bp) was analyzed through reverse transcription on 2% agarose gel. β-actin (294 bp) was used as the housekeeping gene for normalization. (**B**) Western Blotting analysis of HIF1-α (93 kDa) in wild-type and dCAT cells. β-actin (44 kDa) served as the housekeeping gene. (**C**) Densitometric analysis using ImageJ of HIF1-α protein (n=2). (**D**) Enzymatic activity of catalase in wild-type and dCAT cells (n=3). Catalase activity of wild-type cells increased in hypoxia, while no noticeable changes were noted in the catalase activity of dCAT cells under hypoxia.

Table S2. Primers used for guideRNA (gRNA) synthesis and plasmid vector confirmation.

Purpose	Primers	Sequences
gRNA synthesis	Forward	5' – CCCGTGGAGAACCGAACTGCGAT – 3'
	Reverse	5' – AACATCGCAGTTCGGTTCTCCAC – 3'
gRNA fragment amplification	Forward	5' – ACTCTATTCTTCTGTTAGAGGCAACCAC – 3'
	Reverse	5' – TTGACGACTTCTAGCCTCACTCACCTCC – 3'
Sequence analysis	Forward	5′ – TTGGAGAAGTGCTTGCTTCC – 3′
	Reverse	5′ – ACCATCATTTTCTCTTGTCACCCAGGTG – 3′
